# Factors Associated With Health‐Related Quality of Life in Ghanaian Children and Adolescents With Type 1 Diabetes Mellitus: A Cross‐Sectional Study

**DOI:** 10.1002/hsr2.72435

**Published:** 2026-04-28

**Authors:** Benjamin Abaidoo, Akye Essuman, Vera Adobea Essuman, Naa Naamuah Tagoe, Josephine Akpalu, Nana Ama Barnes, Lauretta Pinamang Owusu‐Asare, Yaw Akye Essuman, Vera Mawusime Beyuo, Imelda Ofori‐Adjei, George Awuku Asare, Winfried M. Amoaku

**Affiliations:** ^1^ Ophthalmology Unit, Department of Surgery University of Ghana Medical School, University of Ghana Accra Ghana; ^2^ Department of Internal Medicine University of Health & Allied Sciences Ho Ghana; ^3^ Eye Department Korle Bu Teaching Hospital Accra Ghana; ^4^ Department of Medicine and Therapeutics University of Ghana Medical School, University of Ghana Accra Ghana; ^5^ Santa Rosa Community Health, Vista Clinic Santa Rosa California USA; ^6^ Korle Bu Teaching Hospital Accra Ghana; ^7^ Chemical Pathology Unit, Department of Medical Laboratory Sciences University of Ghana School of Biomedical and Allied Health Sciences Accra Ghana; ^8^ Ophthalmology and Visual Sciences (DCN) University Hospital, QMC Nottingham Nottingham UK

**Keywords:** adolescents, children, quality of life, risk factors, self‐reported, T1DM

## Abstract

**Background and Aims:**

Diabetes is a significant disease that affects individuals of all ages and may lead to the development of systemic and ocular complications, ultimately resulting in a poor quality of life (QoL). The use of health‐related quality of life (HRQoL) measures is important for identifying disparities and those at risk, thereby promoting early intervention. This study aimed to examine the HRQoL and associated factors in Ghanaian children and adolescents with type 1 diabetes mellitus (T1DM).

**Methods:**

A cross‐sectional study involving children and adolescents with T1DM aged 5–19 years was conducted. Demographic and clinical data of participants were recorded. Participants completed the PedsQL Generic Scales questionnaires. SPSS Version 25.0 was used in analyzing the data. Logistic regression was used in analyzing risk factors associated with poor QoL. *p*‐values < 0.05 were considered statistically significant.

**Results:**

Data from 46 children and adolescents with T1DM were analyzed. A female preponderance of 35/46 (76.1%) was observed. The overall mean HRQoL score for participants was 73.9 ± 18.7. Sex was the only risk factor associated with poor self‐reported HRQoL in children and adolescents with T1DM. Female children and adolescents with T1DM were 13 times more likely to have poor self‐reported HRQoL compared with their male counterparts (OR = 13.2; 95% CI = 1.9–91.0; *p* = 0.009). There were no significant associations with age, duration of diabetes, glycemic control, number of hypoglycemic and diabetic ketoacidosis episodes, hypertension, or nephropathy.

**Conclusion:**

Self‐reported QoL of children and adolescents with T1DM was poor. Female children and adolescents with T1DM are more likely to have poor self‐reported QoL.

## 1 Background

Diabetes mellitus (DM) is a multisystemic condition with debilitating effects on the kidneys, nerves, and eyes [1, 2, 3]. In pediatric and adolescent populations, the predominant form of diabetes is type 1 diabetes mellitus (T1DM) [4], with an annual incidence rate of approximately 20 per 100,000 youths in the United States [5, 6].

Most cases of T1DM are characterized by a T cell‐mediated destruction of the pancreatic β‐cells [7]. Children and adolescents with T1DM are often confronted with the risk of developing acute complications such as hypoglycemia and diabetic ketoacidosis (DKA), resulting in morbidities and mortalities [8]. As a result, intensive management of the condition is required, which usually disrupts the daily activities of affected individuals. This demands disease‐focused behavior from the child, which affects the overall quality of life (QoL) [9, 10, 11, 12, 13, 14]. Subsequently, the need for coordinated participation in the management of the condition by these children and their caregivers throughout their developmental stages, from childhood to adolescence, is essential for good glycemic control [15, 16]. Children and adolescents with T1DM are required to embrace a new lifestyle that involves strict adherence to management protocols, including strict dietary regimes.

The assessment of psychosocial factors affecting patients with T1DM using health‐related quality of life (HRQoL) indices is important in identifying children and adolescents at risk of the debilitating effects of DM [17]. Experiences on beliefs, perceptions, and expectations may influence the physical, social, and psychological aspects of the health of a patient [18, 19]. Improving the QoL of these children is essential for good metabolic control in the prevention of complications [20]. Indeed, available evidence supports the importance of achieving a good QoL and glycemic control in the management of children and adolescents with diabetes [20, 21, 22].

Factors such as poor glycemic control, lower education, poor economic status, severe chronic complications, longer duration of diabetes, and sex are associated with poor QoL [23, 24, 25]. Elsewhere, studies have documented the self‐reported QoL in children and adolescents with T1DM [20, 22]. However, such data are limited in developing countries, including Ghana, where access to quality healthcare is poor and DM‐related morbidities are on the increase. This, therefore, led to the quest to examine the HRQoL and associated factors in Ghanaian children and adolescents with T1DM. It is hoped that these findings will support strategies designed to improve the management of children and adolescents with T1DM and reduce the burden of non‐communicable diseases.

## 2 Methods

This study uses data from the same original longitudinal cohort as our previous publications, which reported the full study protocol [26] and ocular changes in children and adolescents with DM at selected facilities [27], using the same study settings and procedures. As such, there is some overlap in the Method sections between the papers. However, these publications address distinct research questions from the current study: while the previous papers focused on the morbidity and complications of DM and ocular changes in children and adolescents with DM – both T1DM and type 2 diabetes mellitus (T2DM) – this manuscript investigates the HRQoL in children and adolescents with T1DM only, exploring influencing factors.

### 2.1 Study Design and Setting

This was a cross‐sectional study conducted at the out‐patient clinics of the Departments of Child Health, Medicine and Therapeutics, Family Medicine, Ophthalmology, and the National Diabetes Management and Research Center (all at the Korle Bu Teaching Hospital, Accra), and Cape‐Coast Teaching Hospital (CCTH) in the Central Region of Ghana from March 2016 to September 2019.

### 2.2 Study Population

Children and adolescents with T1DM aged 5–19 years who attended these clinics during the study period were recruited. Subjects with diabetes diagnosed before 5 years or after 19 years of age and those with T2DM were excluded.

### 2.3 Data Collection and Study Procedure

Patients' demographic characteristics, past medical history, features of systemic complications, including nephropathy, cardiac, neurological, peripheral vascular disease, leg ulcers, and other co‐morbid conditions, were recorded from case notes. Laboratory investigations (fasting plasma glucose, glycated hemoglobin [HbA1c] at diagnosis, hemoglobin [Hb] levels, glutamic acid decarboxylase [GAD] antibodies, Islet cell antibodies, levels of C‐peptide, and lipid profile), blood pressure, and clinical evaluation to identify the presence of microvascular and macrovascular complications were performed and recorded on a pre‐designed data collection form. The blood pressure (lying and standing) was documented. Hypertension was defined as systolic or diastolic blood pressure greater than or equal to the 95th centile for age, sex, and height.

Diagnosis of T1DM was made in patients with classic symptoms such as polyuria, polyphagia, polydipsia, and a random plasma glucose of ≥ 11.1 mmol/L and was further characterized by the presence of GAD antibodies, Islet cell antibodies, and low levels of C‐peptide (stimulated C‐peptide values of < 0.6 pmol/mL) [28, 29, 30]. In this study, patients with HbA1c above the recommended values for age (i.e., < 7.5% for children/adolescents less than 18 years old) by the American Diabetes Association were said to have poor glycemic control [31].

The PedsQL 4.0 Generic Core Scales were used to assess self‐reported QoL outcomes in study participants. The tool contained questions about the participants' physical, emotional, social, and school functioning in the past month. It was a 5‐point Likert scale ranging from 0 (Never) to 4 (Almost always) without the weighting of items on the scale. Permission was sought from the Mapi Research Trust (Lyon, France) for the use of the inventories. Each questionnaire took approximately 5–10 min to complete.

### 2.4 Statistical Analysis

All statistical analyses were performed using SPSS for Windows, version 25.0 (SPSS Inc., Chicago, Ill., USA). Descriptive statistics were reported as means ± standard deviation (SD). The QoL outcomes of Ghanaian children and adolescents with T1DM were assessed using the PedsQL 4.0 Generic Core Scales inventory.

In scoring the process, items on the inventory were reverse‐scored and linearly transformed to a 0–100 scale as follows: 0 = 100, 1 = 75, 2 = 50, 3 = 25, 4 = 0. Where more than 50% of the items in the scale were missing, the scale scores were not computed. Where 50% or more of the items were completed, the mean of the completed items in the scale was computed by summing the items over the total number of items answered. The Psychosocial Health Summary Score was obtained by summing the items across the number of items answered in the Emotional, Social, and School Functioning Scales. The Physical Health Summary Score is the score of the Physical Functioning Scale. The total score was finally obtained by summing all the items over the number of items answered on all the scales. Overall QoL scores were categorized as “good/better” (80%–100%) and “poor” (< 80%).

A two‐sided Chi‐square test or Fisher's exact test was used to examine the association between demographic characteristics and self‐reported QoL outcome. Logistic regression analysis was used to analyze risk factors associated with poor QoL. Variables such as age, sex, body mass index (BMI) category, education, glycemic control, duration of diabetes, hypoglycemic episodes, DKA episodes, and hypertension were entered simultaneously into the logistic regression model. We excluded coronary artery disease (CAD) and nephropathy due to limited cases, which could compromise the stability of the model. Odds ratios (ORs) and 95% confidence intervals (CIs) were recorded and *p*‐values < 0.05 were considered statistically significant.

### 2.5 Ethical Considerations

Written informed consent or assent, as appropriate, was obtained from each participant or guardian. Ethics approval was obtained from the Ethical and Protocol Review Committee (EPRC) of the University of Ghana Medical School with Protocol Identification Number: MS‐Et/M.12‐P4.5/2013‐2014. Permission was also obtained from all the participating institutions. The study followed the tenets of the Declaration of Helsinki.

## 3 Results

### 3.1 Socio‐Demographic and Clinical Characteristics of Children and Adolescents With T1DM

Data from 46 children and adolescents with T1DM were analyzed. A female preponderance of 35/46 (76.1%) was observed. Tables 1 and 2 present the demographic and clinical characteristics of the study participants. The mean age of the participants was 14.4 ± 2.8 years, the minimum age was 5 years, and the maximum age was 19 years. Most participants (34/46, 73.9%) were aged between 13 and 19 years. Most (19/46, 41.3%) were in their Junior High School (JHS) level of education. The majority (29/46, 63.0%) had a normal BMI.

**Table 1:** Socio‐demographic characteristics of children and adolescents with type 1 diabetes mellitus in selected health facilities in Ghana.

Characteristic	Frequency	Percentage (%)
Age groups (years)		
5–7	1	2.2
8–12	11	23.9
13–19	34	73.9
Sex		
Male	11	23.9
Female	35	76.1
Education		
Primary	9	19.5
Junior High School	19	41.3
Senior High School	17	37.0
Tertiary	1	2.2
BMI category		
Underweight	12	26.1
Normal	29	63.0
Overweight	5	10.9

*Note:* Mean age = 14.4 ± 2.8, median age = 15.0 years, minimum age = 5 years, maximum age = 19 years. Mean BMI = 19.9 ± 4.1 kg/m^2^, median BMI = 20.1 kg/m^2^. Minimum BMI = 11.1 kg/m^2^, maximum BMI = 29.3 kg/m^2^.

Abbreviation: BMI, body mass index.

**Table 2:** Clinical characteristics of children and adolescents with type 1 diabetes mellitus in selected health facilities in Ghana.

Characteristic	Frequency	Percentage (%)
Duration of diabetes (years)		
< 1	25	54.4
1–5	15	32.6
> 5	6	13.0
Glycemic control (HbA1c)*		
Good	9	20.5
Poor	35	79.5
Hypoglycemia episodes > 3		
Yes	24	52.2
No	22	47.8
DKA episodes > 3		
Yes	20	43.5
No	26	56.5
Hypertension		
Yes	7	15.2
No	39	84.8
Nephropathy		
Yes	3	6.5
No	43	93.5
CAD		
Yes	12	26.1
No	34	73.9

*Note:* Mean duration of diabetes = 1.8 ± 2.2 years, median duration = 0.9 years, mean age at diagnosis = 10.3 ± 3.5 years, minimum age at diagnosis = 1year, maximum years at diagnosis = 15 years.

Abbreviations: CAD, coronary artery disease; DKA, diabetic ketoacidosis.

* There was some missing data for glycemic control (HbA1c).

The mean age at diagnosis of diabetes was 10.3 ± 3.5 years, with a minimum and maximum age at diagnosis of 5 years and 15 years, respectively. Mean duration of diabetes was 21.3 ± 26.8 months. The mean HbA1c was 10.3 ± 2.9%. The mean BMI was 19.9 ± 4.1 kg/m^2^. The mean low‐density lipoprotein cholesterols and high‐density lipoprotein cholesterols were 3.1 ± 0.9 mg/dL and 0.4 ± 0.2 mg/dL, respectively. Most of the participants (35/46, 79.5%) had poor glycemic control (HbA1c). A majority (25/46, 54.3%) were diagnosed with T1DM 1 year or less before they participated in this study. Insulin was the treatment modality for all the participants. In the past year, 24/46 participants (52.2%) had experienced more than three hypoglycemic episodes, and 20/46 (43.5%) had experienced more than three episodes of DKA. Symptoms of nephropathy were found in 3/46 (6.5%) of the participants. Seven (15.2%) had hypertension, and CAD was found in 12/46 participants (26.1%).

### 3.2 HRQoL of Children and Adolescents With T1DM

The majority of study participants had a poor composite mean score for physical, emotional, school, and psychosocial functioning. Social function was the only domain with a good QoL (83.2 ± 18.6). The overall mean score for HRQoL of the children and adolescents with T1DM was a poor score of 73.9 ± 18.7 (Table 3).

**Table 3:** QoL scores of children and adolescents with type 1 diabetes mellitus in selected health facilities in Ghana.

Quality of life domain	Frequency (%)	Mean ± SD	Rating (Good/Poor)
Physical functioning*		77.4 ± 21.2	Poor
Good	22 (50.0)		
Poor	22 (50.0)		
Emotional functioning		66.7 ± 22.2	Poor
Good	9 (19.6)		
Poor	37 (80.4)		
Social functioning		83.2 ± 18.6	Good
Good	26 (56.5)		
Poor	20 (43.5)		
School functioning		67.9 ± 22.9	Poor
Good	12 (26.1)		
Poor	34 (73.9)		
Psychosocial health		72.6 ± 18.5	Poor
Good	18 (39.1)		
Poor	28 (60.9)		
Overall		73.9 ± 18.7	Poor
Good	19 (43.2)		
Poor	25 (56.8)		

Abbreviation: SD, Standard deviation.

* Two missing data for Physical functioning.

### 3.3 Association Between QoL and Socio‐Demographic and Clinical Variables

The outcome of the association between QoL and socio‐demographic and clinical variables is shown in Table 4 in a bivariate data analysis. The only factor associated with HRQoL among participants was sex (*p* = 0.011).

**Table 4:** Association between quality of life and socio‐demographic and clinical variables among children and adolescents with type 1 diabetes mellitus in selected health facilities in Ghana.

Variable	Better QoL, *N* (%)	Poor QoL, *N* (%)	*p* value
Age groups (years)			0.491
5–7		1 (100.0)	
8–12	6 (54.5)	5 (45.5)	
13–19	13 (40.6)	19 (59.4)	
Sex			0.011*
Male	8 (80.0)	2 (20.0)	
Female	11 (32.4)	23 (67.6)	
Education			0.753
Lower	13 (46.4)	15 (53.6)	
Higher	6 (37.5)	10 (62.5)	
BMI category			0.117
Underweight	6 (50.0)	6 (50.0)	
Normal	13 (48.1)	14 (51.9)	
Overweight	—	5 (100.0)	
Duration of diabetes (years)			0.986
< 1	11 (44.0)	14 (56.0)	
1–5	6 (42.9)	8 (57.1)	
> 5	2 (40.0)	3 (60.0)	
Glycemic control (HbA1c)			0.706
Good	4 (50.0)	4 (50.0)	
Poor	14 (41.2)	20 (58.8)	
Hypoglycemia episodes > 3			0.223
Yes	12 (54.5)	10 (45.5)	
No	7 (31.8)	15 (68.2)	
DKA episodes > 3			0.533
Yes	9 (45.0)	11 (55.0)	
No	10 (41.7)	14 (58.3)	
Hypertension			0.657
Yes	3 (42.9)	4 (57.1)	
No	16 (43.2)	21 (56.8)	
Nephropathy			0.495
Yes	—	2 (100.0)	
No	19 (46.3)	22 (53.7)	
CAD			0.294
Yes	4 (33.3)	8 (66.7)	
No	15 (48.4)	16 (51.6)	

Abbreviations: BMI, body mass index; CAD, coronary artery disease; DKA, diabetic ketoacidosis.

* Statistically significant association.

### 3.4 Risk Factors Associated With Poor QoL in Children and Adolescents With T1DM

The logistic regression model presented in Table 5 shows the strength of association between sex, the only significant risk factor, and poor self‐reported QoL in children and adolescents with T1DM (OR = 13.2; 95% CI = 1.9–91.0; *p* = 0.009). Thus, female children and adolescents with T1DM are 13 times more likely to have poor self‐reported QoL compared with their male counterparts. There was no significant association with age, duration of diabetes, glycemic control, number of hypoglycemic and DKA episodes, hypertension, or nephropathy.

**Table 5:** Risk factors associated with poor QoL in children and adolescents with type 1 diabetes mellitus in selected health facilities in Ghana.

Risk factors	OR	95% CI	*p* value
Age groups (years)			
< 13	Reference	—	—
13–19	0.9	0.1–6.3	0.930
Sex			
Male	Reference	—	—
Female	13.2	1.9–91.0	0.009*
Education			
Higher	Reference	—	—
Lower	1.7	0.3–10.7	0.578
Duration of diabetes			
Long	Reference	—	—
Short	0.7	0.1–5.0	0.750
BMI			
Non‐obese	Reference	—	—
Obese	0.9	0.7–1.2	0.542
Glycemic control			
Poor	Reference	—	—
Good	1.8	0.3–10.7	0.543
Hypoglycemia episodes > 3			
No	Reference	—	—
Yes	2.4	0.4–14.6	0.326
DKA episodes > 3			
No	Reference	—	—
Yes	1.3	0.3–5.7	0.741
Hypertension			
No	Reference	—	—
Yes	1.3	0.2–8.7	0.792

*Note:* Duration of diabetes: Short duration = less than 13 months, long duration = 13 months or more. Education: Lower = JHS to primary, higher = SHS to tertiary.

Abbreviations: BMI, body mass index; CI, confidence interval; DKA, diabetic ketoacidosis; OR, odds ratio; QoL, quality of life.

* Statistically significant association.

## 4 Discussion

The metrics of HRQoL are crucial for providing better support for effective management of patients. Globally, the importance of determining the disease‐specific HRQoL assessment as part of comprehensive care of various chronic diseases is being emphasized [32, 33]. The disease experiences in children and adolescents with T1DM vary significantly [34, 35]. It is therefore essential to incorporate patients' self‐reported perspectives into the management of their disease.

The results of this study revealed a poor overall QoL among these Ghanaian children and adolescents with T1DM. This finding aligns with similar studies conducted in Africa and Asia on children and adolescents with T1DM [36, 37, 38]. The overall poor HRQoL observed in this study may be attributed to several factors. These include children and adolescents with T1DM facing various challenges such as adhering to treatment regimes, managing chronic conditions, and coping with the emotional and psychosocial effects associated with the disease [14, 39, 40, 41]. Similar to what is observed elsewhere in Africa [36, 37, 38, 42], reduced school functioning may be linked to frequent school absenteeism due to hospital appointments and admissions [43]. Interventions that allow more flexible school schedules to support disease management would assist children and adolescents to adjust to their condition while minimizing interruptions to their social interactions.

Poor emotional and psychological health may also stem from stress and anxiety related to the disease itself and its long‐term complications [40, 41]. Healthcare providers serving children and adolescents with T1DM should be vigilant to identify children who exhibit signs of mental health disorders such as depression and anxiety, and offer appropriate support. Psychologists specialized in pediatric care should be part of the multidisciplinary care team and available at diabetes clinics to provide support for children and their families as needed.

Social functioning, however, showed better mean scores, corroborating findings from the study by Bekele et al. [42] in Addis Ababa. This could be attributed to the strong extended family structure and cultural emphasis on community and collective well‐being in Ghana [44, 45]. Ghana's strong extended family structure and cultural emphasis on community have important implications for the HRQoL of children and adolescents living with T1DM. On one hand, close family and community networks may provide substantial emotional, practical, and financial support, including shared caregiving responsibilities, encouragement with treatment adherence, and assistance with healthcare costs, all of which can enhance psychosocial well‐being and reduce feelings of isolation in children. On the other hand, deeply rooted cultural beliefs and social expectations within extended families and communal settings may sometimes pose challenges to optimal diabetes self‐management, such as promoting non‐biomedical explanations of illness, pressure to conform to communal eating practices, or difficulties navigating disclosure and stigma. Together, these supportive and potentially constraining influences highlight the complex role of Ghana's sociocultural context in shaping HRQoL outcomes among young people with T1DM and underscore the importance of culturally sensitive, family‐centered diabetes care.

Our finding that female sex (OR: 13.2; 95% CI: 1.9–91.0) was the only risk factor significantly associated with poor self‐reported HRQoL in children and adolescents with T1DM aligns with those of other studies, which have shown that females with T1DM tend to report lower QoL than males [33, 46] This lower QoL in females with T1DM may be attributed to a combination of hormonal, psychosocial, cultural, and healthcare‐related factors [47]. Research indicates that females, especially adolescents, are at a higher risk of mental health issues such as depression and anxiety [48, 49]. The added pressure of maintaining stable blood glucose levels may further worsen the emotional burden on females [50]. Additionally, hormonal changes associated with the menstrual cycle in adolescents can adversely impact the management of glucose levels [51, 52, 53]. Such fluctuations can increase stress and emotional strain, ultimately leading to a negative impact on their overall QoL. Although female sex was identified as a significant predictor of poor HRQoL, the OR was associated with a wide confidence interval, likely reflecting the relatively small sample size and the imbalance in the number of male and female participants. Therefore, the magnitude of this association should be interpreted with caution, and further studies with larger and more balanced samples are needed to confirm this finding.

The results of this study did not show any significant association with other factors, including age, glycemic control, and duration of the disease, although some studies have reported lower QoL with increasing age [37, 42]. For example, Bekele et al. [42] reported lower scores for the emotional functioning subdomain with increasing age, attributing this to the fact that younger children typically received more direct care from their parents compared to adolescents. Additionally, it is natural for adolescents to worry more about their disease condition compared to younger children, which may contribute to the lower scores observed in adolescents. Bekele et al also found that a longer duration of diabetes and less frequent glucose monitoring were associated with lower HRQoL scores [42]. This may be attributed to the increased number of hospital visits, the discomfort associated with frequent repeated blood tests, and the prolonged duration of treatment that often accompanies these factors.

Other studies have demonstrated better HRQoL scores in children and adolescents with well‐controlled glucose levels [42, 54, 55]. This was attributed to a reduced incidence of common acute complications of diabetes, such as hypoglycemia, hyperglycemia, and DKA. It has also been suggested that well‐controlled blood glucose levels are linked to improved cognitive function and better academic performance [56, 57]. No such association was demonstrated in the present study.

Studies comparing HRQoL self‐reports from children and adolescents to parent proxy reports also found significant differences in their results [38, 42, 58, 59]. These studies reported lower HRQoL in proxy parent reports compared with self‐reports by children and adolescents. The differences in scores were attributed to the perceptions of primary caregivers, which tend to be more influenced than those of their children [59]. Since primary caregivers typically bear greater responsibility for a child's health, they are more likely to perceive a higher impact on the child's QoL, which may result in reporting a lower HRQoL.

The diagnosis of T1DM is associated with significant physical and psychosocial challenges for children, adolescents, and their families. These children and adolescents face unique challenges that can impact their QoL. It is suggested that targeted interventions should focus on addressing the risk factors in a multifaceted approach to improve QoL in children and adolescents with T1DM. Such interventions should integrate psychological, medical, and social support in their care, and may include multidisciplinary care teams, family involvement and education, psychosocial interventions, and the integration of technological innovations.

The diagnosis and management of T1DM in children and adolescents imposes substantial physical and psychosocial burdens on both patients and their families, with parents frequently experiencing elevated stress, anxiety, and depression due to constant monitoring, fear of complications, and responsibility for treatment adherence [60]. This parental distress can influence family dynamics and the HRQoL of both children and households [60]. Family‐focused interventions, including weekend‐based parent group programs, can significantly reduce parenting stress by enhancing self‐efficacy, social functioning, and perceived competence in managing the child's condition [61]. Research in this area indicates that effective T1DM management extends beyond medical treatment to a family‐centered approach, where integrated clinical care and structured psychosocial support are essential for promoting adaptive coping, improving HRQoL, and enhancing long‐term outcomes for children and their families [61].

This study has several limitations that should be considered when interpreting the findings. First, the relatively small sample size (*n* = 46) may have limited the statistical power to detect moderate associations between HRQoL and some clinical variables such as age, duration of diabetes, glycemic control, and complications. Consequently, the absence of statistically significant associations for these variables should be interpreted cautiously. Second, there was a marked gender imbalance, with females comprising 76.1% of participants, which may have influenced the observed gender differences in HRQoL and potentially reflected gender‐specific health‐seeking behaviors rather than true population‐level differences. Third, participants were recruited from selected health facilities, which may not fully represent all children and adolescents living with T1DM in Ghana and may have introduced selection bias, as individuals with regular access to specialized care may differ systematically from those with limited access to healthcare services. Finally, the study relied on self‐reported data, which are subject to recall and social desirability biases and may have affected the accuracy of participants' responses. Although validated instruments and standardized data collection procedures were used to minimize these biases, they cannot be entirely excluded. Consequently, the findings should be interpreted cautiously and viewed as exploratory. Future studies with larger sample sizes, more balanced gender representation, broader population‐based or multicenter recruitment, and the inclusion of objective clinical measures are warranted to enhance representativeness and external validity. Another limitation of our study is that data on socioeconomic status, family structure, or access to healthcare were not collected. These factors are known to influence HRQoL and may act as confounders. We recommend that future studies incorporate these variables to provide a more holistic evaluation of HRQoL in children and adolescents with T1DM in Ghana.

Notwithstanding, the utilization of validated tools for assessing the QoL of participants in this study provides robust evidence for risk factors associated with poor QoL in children and adolescents with T1DM. As such, the results from this study may contribute to the management of T1DM.

## 5 Conclusions

Findings from this study show that the self‐reported HRQoL of Ghanaian children and adolescents with T1DM is poor. Female children and adolescents with T1DM are more likely to have poor self‐reported QoL. Multidisciplinary care teams should be established for children and adolescents with T1DM to address their physical, psychological, social, and emotional well‐being.

## Author Contributions

Conception and design: Akye Essuman, Benjamin Abaidoo, Vera Adobea Essuman, and Winfried M. Amoaku. Data acquisition: Akye Essuman, Benjamin Abaidoo, Josephine Akpalu, Vera Adobea Essuman, George Awuku Asare, Naa Naamuah Tagoe, Imelda Ofori‐Adjei, Vera Mawusime Beyuo, and Nana Ama Barnes. Data analysis and interpretation: Benjamin Abaidoo, Akye Essuman, Vera Adobea Essuman, Winfried M. Amoaku, and Josephine Akpalu. Writing – Initial draft: Benjamin Abaidoo and Akye Essuman. Funding acquisition: Vera Adobea Essuman. All authors have read and approved the final version of the manuscript. Akye Essuman had full access to all data in this study and takes full responsibility for the integrity and accuracy of the data analysis. All authors have agreed both to be personally accountable for their own contributions and to ensure that questions related to the accuracy or integrity of any part of the work, even ones in which they were not personally involved, are appropriately investigated, resolved, and the resolution documented in the literature.

## Disclosures

Support from the University of Ghana Research Fund (URF/9/LMG‐013/2015‐2016).

## Ethics Statement

Ethics approval was obtained from the Ethical and Protocol Review Committee (EPRC) of the University of Ghana Medical School with Protocol Identification Number: MS‐Et/M.12‐P4.5/2013‐2014. Permission was also obtained from all the participating institutions. The study followed the tenets of the Declaration of Helsinki.

## Consent

Written informed consent or assent, as appropriate, was obtained from each participant or guardian.

## Conflicts of Interest

The authors declare no conflicts of interest.

## Transparency Statement

The lead author, Benjamin Abaidoo, affirms that this manuscript is an honest, accurate, and transparent account of the study being reported; that no important aspects of the study have been omitted; and that any discrepancies from the study as planned (and, if relevant, registered) have been explained.

## Data Availability

The datasets used and/or analyzed during the current study are available from the corresponding author on reasonable request.
